# Why patients stay in or leave a medically tailored meals program: a qualitative study

**DOI:** 10.3389/fpubh.2026.1737511

**Published:** 2026-02-06

**Authors:** Sara C. Folta, Jessica Burch, Matthew Alcusky, Arlene S. Ash, Kurt Hager, Jean Terranova, Fang Fang Zhang, Aoife O’Flaherty, Oyedolapo Anyanwu, Dariush Mozaffarian

**Affiliations:** 1 Food is Medicine Institute, Friedman School of Nutrition Science and Policy, Tufts University, Boston, MA, United States; 2 Community Servings, Jamaica Plain, MA, United States; 3 T.H. Chan School of Medicine, University of Massachusetts, Worcester, MA, United States

**Keywords:** enrollment, food is medicine, medically tailored meals, qualitative, withdrawal

## Abstract

**Introduction:**

Medically tailored meals (MTMs) are home-delivered, nutritionally tailored meals designed for patients with complex or advanced diet-sensitive medical conditions and social stressors. Although MTM use can improve food security, diet quality, and health outcomes, and reduce overall healthcare use and cost, little is known about why patients enroll in, stay in, or withdraw from such programs.

**Methods:**

Between June 2023 and May 2024, we explored factors related to MTM program completion or withdrawal using semi-structured qualitative interviews among 28 patient participants in a program run by the non-profit Community Servings. Half had completed the 6-month program (“completers”), and half had requested early discontinuance (“non-completers”). The interviews covered patient factors (health status, health goals, motivation to participate) and program characteristics (perceptions of the program overall, logistics, meal characteristics). Interviews were recorded and transcribed, and then coded using NVivo software. We used directed qualitative content analyses and included matrix coding queries to compare themes overall and between the groups.

**Results:**

Both completers and non-completers described enrolling to alleviate symptoms, regain physical function, and engage in desired activities. Many non-completers also focused on weight loss. Before joining, non-completers had been more enthusiastic about changing their diets, while completers were more interested in alleviating financial strain and the time and physical challenges associated with meal preparation. Both groups had very positive perceptions of the program. Both groups initially found the meals bland and portion sizes small, but completers more readily adapted to both taste and portion size. In contrast, among non-completers, taste was a reason for discontinuation for some. Other non-completers withdrew for “good reasons”: they felt better or their circumstances otherwise changed, making the meals seem unnecessary. Both groups found the experience of eating the meals to be educational, which supported sustained dietary changes.

**Discussion:**

These novel findings explore patients’ reasons for starting, completing, and stopping MTMs. Findings suggest strategies to improve program completion, such as addressing expectations about weight outcomes, taste, and portion size. Our study demonstrates the value of patient feedback for learning how to improve program effectiveness.

## 1 Introduction

“Food is Medicine” programs, in which food and nutrition are integrated into healthcare delivery, have shown promise for improving nutritional and health outcomes and equity in several studies (1). Medically tailored meals (MTMs) are one such program that is expanding in the U. S., for example, through Medicaid Section 1115 Demonstrations, in lieu of services, and Medicare Advantage programs (2). MTM programs provide fully prepared, nutritionally tailored meals for individuals living with complex or advanced diet-sensitive medical conditions such as diabetes, heart failure, end-stage renal disease, HIV/AIDS, or cancer. Recipients often have social stressors and complex unmet needs, for example, related to food insecurity, low income, comorbid mental health issues, and/or activity limitations. Meals are home-delivered and designed by a registered dietitian nutritionist and culinary team based on the medical diagnosis and a nutritional assessment (3). A growing body of research supports the effectiveness of MTMs in improving food security, diet quality, and health outcomes (4–7) and in reducing healthcare utilization and costs (4, 8, 9). However, another critical aspect of achieving beneficial outcomes is patients’ willingness and ability to enroll and stay in the program.

Studies of MTM programs to date have found a wide range of patient withdrawal rates, from 1–5% (10–12) to 17–42% (6, 13–15). These studies included patients with heterogeneous medical conditions and programs of varying lengths, from 4 weeks to 6 months (6, 10–15). Across the studies, program duration appears unrelated to withdrawal, with the shortest program (4 weeks) having a high withdrawal rate (32.6%) (15), while a 6-month program had a very low withdrawal rate (1%) (10). Similarly, neither the medical conditions addressed nor the source of the meals (non-profit or for-profit partners) show a discernible pattern with the frequency of withdrawal. Reasons for program withdrawal were uncommonly solicited and reported, and when they were, included disliking the food, a preference for their own food, gastrointestinal reactions, illness, or a perception that the portions were too large (11–13, 15). Thus, the reasons for patient continuation or withdrawal from MTM programs remain unclear and understudied.

Understanding and addressing this knowledge gap is critical for the success of MTM programs, especially as more U.S. states and other payers begin to support MTM. It is also crucial to understand and value patient experiences, needs, and preferences as MTM programs are designed, refined, and scaled. However, to our knowledge, few studies have explored in depth the patient perceptions related to continuation or withdrawal from MTM programs, as well as the initial patient perceptions contributing to enrollment. To gain these insights, we conducted semi-structured qualitative interviews with patients who enrolled in a six-month MTM program, including both those who completed and those who chose to withdraw from the program.

## 2 Methods

### 2.1 Setting, program, and sample

**Figure 1:**
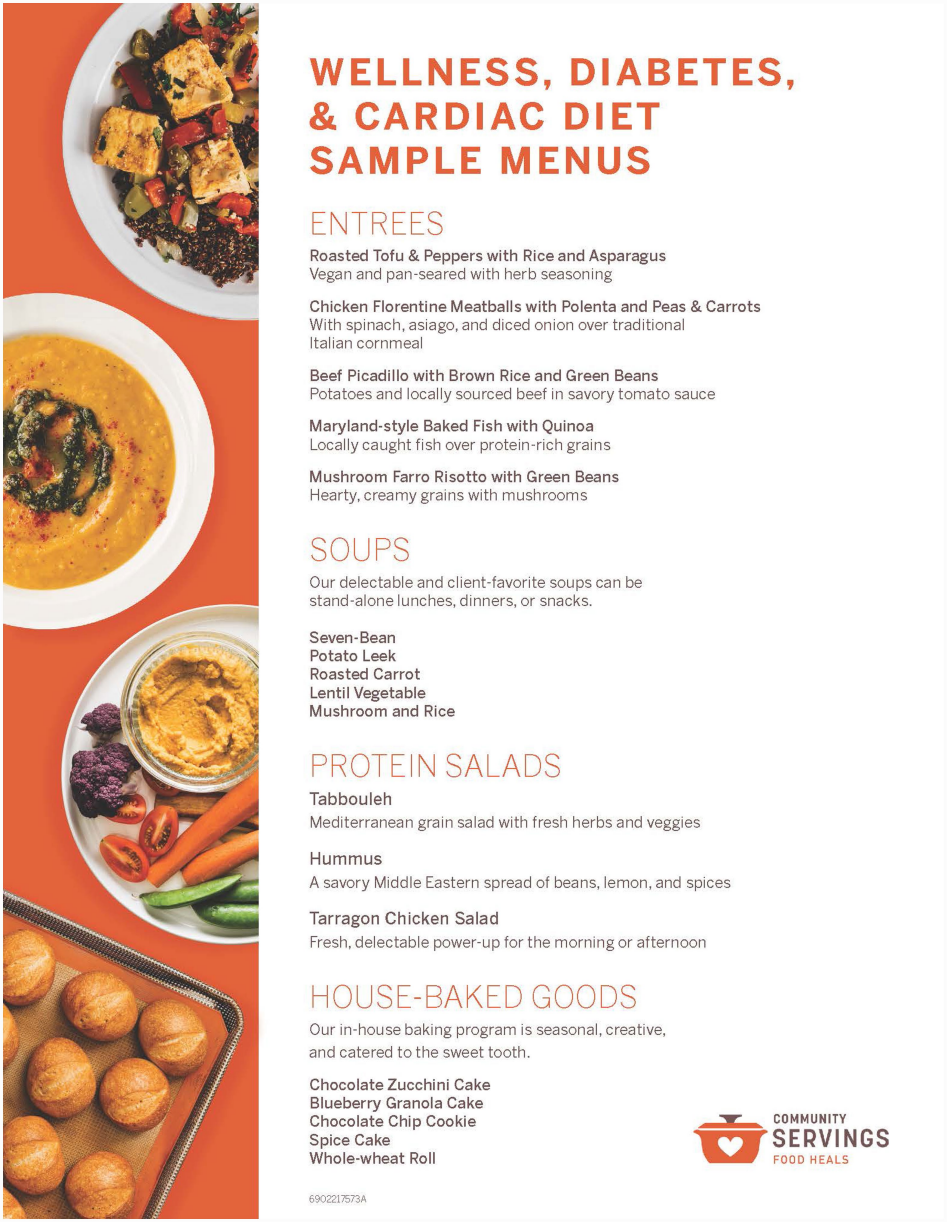
Example Community Servings menu combining three core diets (wellness, diabetes, and cardiac).

**Figure 2:**
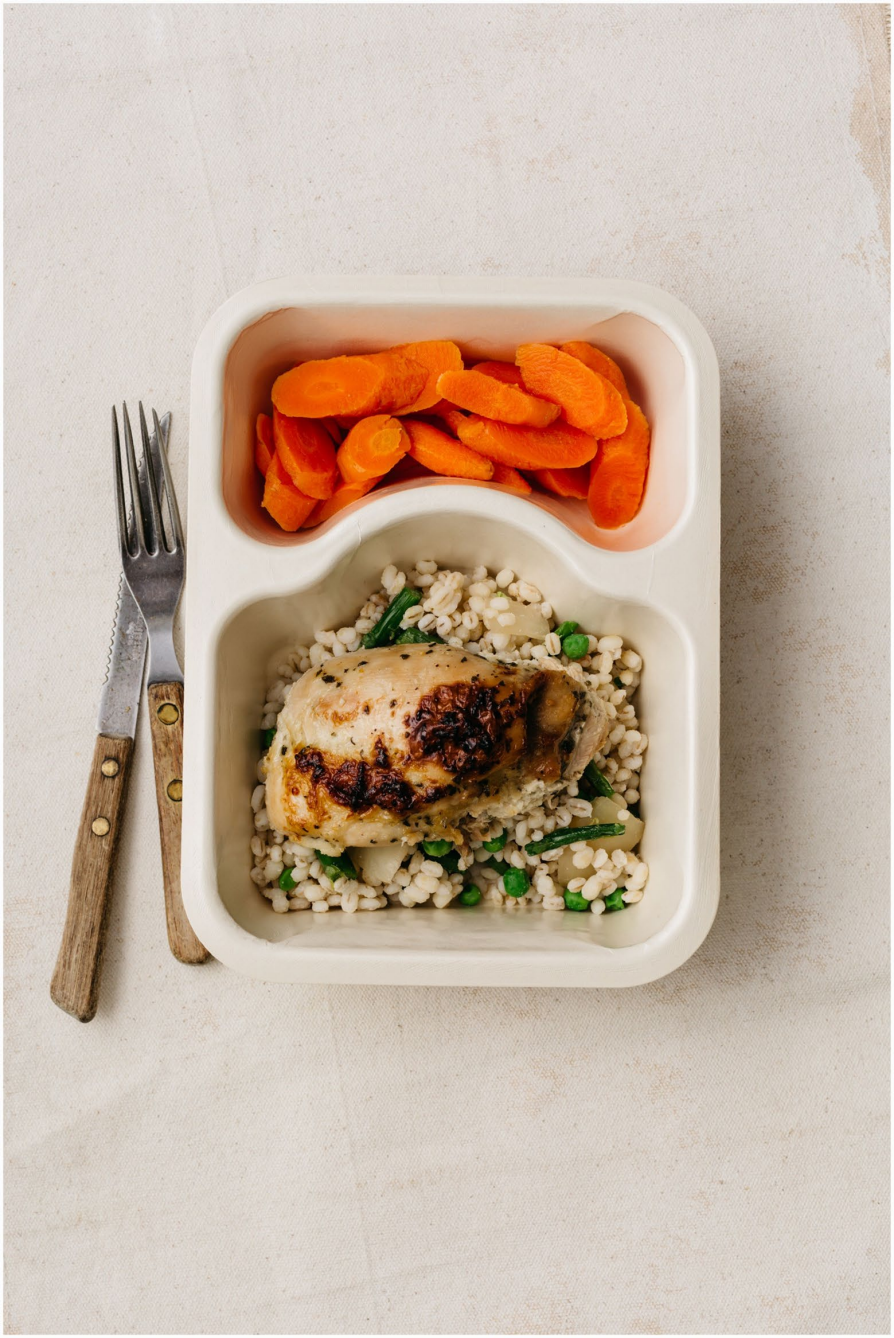
Example of a Community Servings meal.

We enrolled patients participating in an MTM program provided by Community Servings, a non-profit organization in Massachusetts with state-wide reach and over 30 years of experience in providing home-delivered MTMs. The meals provided by Community Servings are designed by registered dietitian nutritionists (RDNs) and a culinary team to meet Food is Medicine Coalition (FIMC) accreditation standards for medically tailored meals (FIMC accreditation criteria and requirements are available at https://fimcoalition.org/wp-content/uploads/2024/03/Food-is-Medicine-Coalition-Medically-Tailored-Meal-Intervention-Accreditation-Criteria-and-Requirements.pdf). More specifically, Community Servings meals provide approximately two-thirds of the total calories of a 2,000 kilocalorie diet. Thus, each weekly delivery provides roughly 1,340 kilocalories per day in the form of scratch-made dinners (5 per week), soup and protein-based salad lunch items (5 per week), snacks, desserts, and milk, with a preference for sourcing from local farms whenever possible. Sodium and saturated fat levels are standardized according to evidence-based dietary patterns, with sodium limits of 500 mg for entrees and 400 mg for soups. Total saturated fat is no more than 10% of calories provided (less than 9 g of saturated fat per day). Meals are tailored to meet a variety of dietary needs and preferences, with 16 diet offerings, including six core diets and 10 modifiers. The six core diets are: wellness, pediatric, renal, diabetic, cardiac, and maternal health. The modifiers include mild, soft, low fiber, pescatarian, low lactose, vegetarian, no fish, no red meat, high calorie/protein, and no nuts. During an initial intake consultation with an RDN, participants may combine up to three diets to tailor the intervention to their individual needs. Example combinations might include soft and mild for someone with acid reflux who needs carbohydrate-controlled, easy-to-chew meals; or maternal health and no fish, for someone with increased energy demands during pregnancy who does not like fish. An example menu of a common combination of three core diets -- wellness, diabetes, and cardiac -- is provided in Figure 1, and an example photo of a meal is provided in Figure 2. All meals contain the same portion sizes. The meals are fully prepared and only need to be heated up. As part of the program, participants are offered three sessions with an RDN. The MTMs were generally offered for 6 months, with the opportunity to re-enroll if recommended by a patient’s care team.

Throughout its history, Community Servings has collected feedback on its services through participant surveys. Based on this feedback, it has adapted its meals over time, for example by adding or eliminating specific food items and dietary modifiers as well as modifying packaging to prevent spilling/cracking of containers and to ensure optimal temperature control. They have also responded to participant feedback by offering information, such as suggestions for seasoning meals without salt. However, Community Servings has collected much less in-depth, qualitative feedback, which may serve to complement and extend quantitative survey data; this type of feedback has also not been widely reported in the literature on MTMs.

A purposive sampling strategy was used for this study. “Completers” included those who either stayed in the program for the initial 6 months and were interviewed within the next 3 months after completion (*n* = 10), or those who had been renewed for a second 6 months and were interviewed within 3 months of the renewal period ending (*n* = 4). “Non-completers” were those who withdrew from the program before the six-month period ended by specifically requesting to no longer receive the meals (*n* = 14) and were interviewed within 3 months of withdrawal. Other eligibility criteria for this analysis included being an adult (age 18–65 years) and having both food insecurity and a qualifying medical condition, including but not limited to diabetes, cardiovascular disease, high-risk pregnancy, or behavioral health conditions. The exclusion criterion was refusal to allow the interview to be recorded. Initially, participants were recruited from among those whose participation was supported under Massachusetts’ Medicaid 1,115 Demonstration waiver, which covered MTMs and other nutrition services for qualifying members with food insecurity and significant health issues who were enrolled in Medicaid accountable care organizations (ACOs). Recruitment was initially conducted through two ACOs that granted Community Servings permission to invite their enrollees to participate in the research study. However, it was challenging to recruit non-completers who were less numerous and harder to contact. Consequently, patients whose participation was supported by philanthropy were also recruited, most of whom had also been referred by their healthcare team. All potentially eligible patients were identified by Community Servings by reviewing patient lists. A Community Servings intern, also a student at the Friedman School of Nutrition at Tufts University (AO), reached out by phone, email, or text to determine patient interest in participating. Of 107 patients approached about participation, 28 agreed to participate and completed an interview. Reasons for non-participation included inability to contact (*n* = 37), declining the interview (*n* = 30, no further reasons given), and inability to schedule the interview after initial interest in participation (*n* = 12). The protocol was reviewed and deemed exempt by the Tufts University Health Sciences Institutional Review Board, and verbal informed consent was obtained from all participants.

### 2.2 Interview procedures

The semi-structured interview guide, developed by SCF with input from the research team, was informed by the Health Equity Implementation Framework (16, 17), which prioritizes assessing the impact of implementation on health equity within healthcare settings. To explore similarities and differences between program completers and non-completers, we focused on two of the framework domains: patient factors (health status, health goals, motivation to participate) and characteristics of the intervention (perceptions about the program overall, logistics, meal characteristics). Table 1 presents the interview questions used, along with their corresponding analysis codes and definitions.

**Table 1:** Domains, interview questions, and codes and definitions.

Domain	Interview questions	Codes and definitions
Patient factors	How would you describe your health in general right now? In what ways, if any, does your health affect your everyday quality of life? Tell me about any health-related goals you have for yourself. Thinking back to when you were first told you about the program, what was your reaction to it? Describe how willing you were to try it. What were the reasons for your interest in it? What were your concerns about participating in the program, if any? When you agreed to participate in the program, what were you hoping to get out of it? Talk about how well the program met those expectations. Talk about your interest in changing your diet before you started this program. In your opinion, how important is this type of program in general? As you know, the meals were designed with your medical condition in mind. Tell me about whether you have been able to eat in a similar way since the meals stopped. What has helped you to continue to eat that way? What gets in the way of eating that way?	Health and quality of life: patient’s description of their general state of health Health goals: addresses any health goals the patient has set prior to the program (excludes health expectations specific to program participation) Receptivity: patient’s initial receptiveness to the program, including their level of interest in enrolling Health expectations: patient’s description of any health benefits they expected from participation prior to enrolling Nutrition motivation: patient’s general perceptions about healthy eating and motivation for healthy eating prior to the program Overall value: patient’s thoughts about the perceived importance of the program and why Behavior change sustainability: patient’s description of the extent to which they have maintained a diet similar to the medically tailored one, including motivation to sustain the change Facilitators to sustaining dietary changes: patient’s description of specific facilitating factors to continue healthy eating after the program ended Barriers to sustaining dietary changes: patient’s description of specific barriers to eating healthfully after the program ended
Characteristics of the Intervention: Perceptions about Participation	What problems did you have with getting enrolled in the program, if any? What was your experience with meal delivery? What worked well in terms of the delivery of the meals? What issues did you have with the meal delivery, if any? Tell me what you thought about the meals. Talk about the meals in terms of meeting your needs. What did you think about how the meals tasted? What did you think about the portion sizes? What would you have changed about the meals?	Enrollment: patient’s general perceptions about the referral process for the program, including any issues with enrollment Meal delivery: perceptions of how the meal delivery process worked overall, including specific components of the meal delivery process Meal type: thoughts about the type and quality of the meals, including taste, variety, and portion size Improvement of meals: ideas about how the meals could be improved, including quality, portion size, and variety, or stating that no improvements needed

Interviews were conducted from June 2023 to May 2024 via phone using the Votacall videoconferencing application by the Community Servings intern, who was trained by SCF. Interviews lasted an average of 61 min (65 min for completers, 58 min for non-completers). All interviews were conducted in English. Participants were provided a $25 gift card to remunerate them for their time.

### 2.3 Analysis

All interviews were recorded and transcribed, and then coded using NVivo software (version 12, QSR International, Doncaster, Australia). We used a deductive directed qualitative content analysis approach (18), drafting an initial codebook based on the interview guide. We then reviewed the transcripts and refined the codebook, assigning codes to emerging topics. A research team member (OA) then coded the transcripts. To determine inter-rater reliability, SCF independently coded six randomly selected transcripts: three each from completers and non-completers. A kappa coefficient of 0.7 or greater at each code (substantial agreement) (19) was deemed acceptable. After one child code was merged with a parent code, all codes met the 0.7 threshold.

To compare themes between completers and non-completers, we first examined the data within each group separately. We then compared themes between the two groups through a manual review of the themes from each group. We formally compared the themes using matrix coding queries within NVivo. The themes, including the differences and similarities between completers and non-completers, were discussed by the study team and shared with our partners at Community Servings.

## 3 Results

Characteristics of the 14 completers and 14 non-completers are shown in Table 2. The average age was 51.5 years (range: 25 to 65 years). Sixty-one percent were female; 57%, white; and 25%, Hispanic. Most (86%) were born in the U. S. Employment status varied, with nearly one-third (32%) unable to work. Most (89%) were single. For about two-thirds of participants, program participation was supported by the state Medicaid 1,115 demonstration, and it was supported by philanthropic funding for about one-third. Ten of the non-completers were supported by philanthropic funding, while none of the completers were. The groups were similar on most demographic characteristics.

**Table 2:** Participant demographic characteristics (*N* = 28).

Characteristic	Full sample	Completers (*n* = 14)	Non-completers (*n* = 14)
Age in years,* average (min-max)	51.5 (25–65)	52.5 (35–63)	50.6 (25–65)
Gender female, *n* (%)	17 (60.7)	9 (64.3)	8 (57.1)
Race, *n* (%)			
Black White Other	10 (35.7) 16 (57.1) 2 (7.1)	4 (28.6) 8 (57.1) 2 (14.3)	6 (42.9) 8 (57.1) 0 (0)
Hispanic, *n* (%)	7 (25.0)	4 (28.6)	3 (21.4)
Born in U.S., *n* (%)	24 (85.7)	12 (85.7)	12 (85.7)
Employment status, *n* (%)			
Employed full-time	7 (25.0)	3 (21.4)	4 (28.6)
Employed part-time	5 (17.8)	4 (28.6)	1 (7.1)
Not employed	5 (17.8)	1 (7.1)	4 (28.6)
Unable to work	9 (32.1)	6 (42.9)	3 (21.4)
Student	1 (3.6)	0 (0)	1 (7.1)
Retired	1 (3.6)	0 (0)	1 (7.1)
Marital status, *n*			
Married	3 (10.7)	1 (7.1)	2 (14.3)
Never married	16 (57.1)	6 (42.9)	10 (71.4)
Divorced, separated, or widowed	9 (32.1)	7 (50.0)	2 (14.3)
Program participation support			
Medicaid 1,115 Waiver	18 (64.3)	14 (100)	4 (28.6)
Philanthropic	10 (34.7)	0 (0)	10 (71.4)

^*^n = 26.

### 3.1 Identified themes

Consistent with our deductive approach, we present themes in two broad domains: (1) patient factors, including health status, health goals, and motivation to participate; and (2) characteristics of the intervention, including perceptions about the program overall, perceptions about the meals specifically, and thoughts about changes resulting from participation. Similarities and differences in identified themes between completers and non-completers are described in each domain and summarized in Table 3.

**Table 3:** Summary of findings.

Theme	Completers compared to non-completers
Patient factors	
Health status	In both groups, health issues impacted activities of daily living
Health goals	Patients in both groups wished to alleviate symptoms or regain function, but non-completers mentioned weight loss as a specific focus more often
Motivation for MTM participation	Completers were interested in the program since the meals were free and convenient, and were not especially interested in changing their diet Non-completers were more enthusiastic when they heard about the program and appreciated the alignment between the meals and their health conditions
Characteristics of the intervention: perceptions about participation	
Perceptions about the program overall	Both groups expressed positive attitudes toward the program in terms of their experiences with the logistics and its larger role in helping people with health conditions A reason for withdrawal among non-completers was that they felt better or their circumstances otherwise changed, and several wanted to be sure that others could benefit
Thoughts about the meals	Both groups described the meals as bland, and the portion size seemed too small at first Completers adapted to the meals over time, often growing to like the taste and appreciate the portions as appropriate Many non-completers could not adapt to the taste and withdrew for this reason; some were also not able to adapt to the portion size
Sustaining change after the program	Both groups were motivated to sustain dietary changes Patients in both groups described the experience of the tailored meals as educational in a way that facilitated sustained dietary change, although completers described more specific strategies Among completers, the cost of healthier food was described as a barrier to sustained change

### 3.2 Patient factors: health status, health goals, and motivation to participate

To be eligible, all participants had to have a chronic illness and/or multiple health needs. Both completers and non-completers described multiple ways that their health affected their activities of daily living.

Completer: *“I use a walker to get around. I get very tired easily, so I need help with a lot of things, like cleaning my house, I need help food shopping, I need help going to doctors.” Female, age 61, unable to work*

Non-completer: *“I have my kidney problems. I'm doing dialysis for six years….It affects a little bit. Sometimes I cannot work and I cannot-- Whatever I do, sometimes I get tired and I have to stop. If I have to go upstairs, and sometimes I have to stop and take a break and go up again. Those things.” Male, age 44, not employed*

In describing their health-related goals that motivated participation in the MTM program, completers focused on alleviating symptoms and regaining function to engage in desired activities, including work. While generally sharing similar goals, half of the non-completers also focused specifically on weight loss.

Completer: *“Honestly, the ultimate goal is to actually try to get back out in the workforce. Obviously, I realize that that's going to be a challenge. The reason why I say that is, when people look at my condition, they don't want to take a chance in trying to hire somebody like me…Right now it's just frustrating in the sense where I used to be able to do a lot of things on my own, and I can't do anything by myself anymore. My ultimate goal is to get better and actually start doing something [for work], whether it be part-time, full-time, whatever I can handle.” Male, age 52, unable to work*

Non-completer: *“Well, to lose about maybe a good 50 pounds…My goal is to lose some weight, to eat healthier, that will help me to maintain a healthy weight and help me to do better in general.” Female, age 54, employed part-time*

Completers and non-completers differed in their nutritional motivations to participate in the MTM program. Among completers, many stated that they were not particularly interested in changing their diet, as they had entrenched food preferences and habits.

Completer: *“I was not interested in changing my diet. I was like, ‘Uh.’ I don’t know, I was willing to participate, to try it, and I said, ‘Okay’ but after a few weeks, I started liking it.” Female, age not disclosed, employed full-time*

Completer: *“Now, like I said, I used to eat the same things over and over again when I was shopping for my own stuff because I just didn't like-- I guess afraid of change or afraid of trying different things. I knew what I liked and that's what I got, even though I knew it wasn't good for me.” Male, age 52, unable to work*

However, they were willing to try the program, mainly because of the financial strain, time, and physical challenges of preparing their own meals.

Completer: *“My reaction was, ‘Oh, thank God.’ When I'm exhausted, I don't have to stand and cook. I can just microwave the food and it's ready.” Female, age 61, unable to work*

Completer: *“It's free [chuckles]. Main reason because I could never afford anything else…” Male, age 52, unable to work*

Completer: *“I thought that only people that really needed it [chuckles] would get it. Then I started realizing that it was much easier because some of the meals already just came prepared. All you had to do was heat it up.” Female, age 57, unable to work*

On the other hand, non-completers expressed enthusiasm about the nutritional benefits of starting the program. They remembered thinking it was “wonderful” and a “great idea” to have “healthy meals” and were motivated to participate by the alignment of the nutritional quality of the meals with their health conditions.

Non-completer: *“Also, they mentioned how the meals, they’re balanced meals and stuff, healthy meals pretty much, and healthy snacks. That would [help] with my weight management. I thought it was overall a good idea. I was excited about it because I could finally save money and I was finally going to lose weight.” Female, age 25, student*

Non-completer: *“The things I was worried about was my salt intake and my high blood pressure… My doctor suggested it [MTM] because it had low-sodium meals, which it did.” Male, age 60, not employed*

### 3.3 Characteristics of the intervention: perceptions about participation

Patient perceptions about program participation were identified and grouped into three thematic areas: (1) overall perceptions about the program, including experiences with its logistical aspects and their thoughts about MTMs as a general concept; (2) their experiences with the meals, including taste and portion size; and (3) their ability to sustain dietary changes when no longer receiving the meals.

#### 3.3.1 Overall perceptions of the program

Both completers and non-completers expressed positive attitudes toward the program, both in terms of their experiences with its logistical aspects and its general usefulness. Patients in both groups described the enrollment process and meal delivery as smooth, with few issues reported.

Completer: *“[Staff person responsible for enrolling patients] came to my house. I sat down with her face-to-face so I could actually, if I couldn't hear her, I could read her lips. She did everything for me. We went through everything and that's how they determined what to give me, what not to give me, that type of thing. I had a lot of help with that but when she set it up, it was perfect. It was just, everything was right. It was great.” Male, age 52, unable to work*

Non-completer: *“She put in a referral, I got a callback very soon after, and they did the intake interview. I got the meals the following Wednesday, like when they said that I would get them, and that was it, it couldn't have been easier. [laughs] Usually, you're used to having to sign 17 million forms to get signed up for something like this, I didn't sign one single piece of paper, it was all over the phone and super quick.” Male, age 32, employed full-time*

The delivery schedule worked for patients and was felt to be flexible. Participants had positive impressions of the delivery personnel, who they felt went the extra mile to be helpful and respectful.

Completer: *“The delivery was excellent. I've never had a problem. The guy would call me, and if I couldn't get to the door, he'd leave it outside. I have a screen, like a screen door. He'd put it inside the thing so no one would touch it. It came frozen, so all I had to do was defrost it when I wanted it. It was really an excellent experience. I'm sad that I didn't know about it years ago, because I probably would have joined years ago.” Female, age 55, unable to work*

Non-completer: *“I thought they were very courteous when they called, they would wait until I came to the door and picked it up. They didn't just drive away.” Male, age 59, unable to work*

Both completers and non-completers also endorsed very positive attitudes toward the program’s broader role in supporting individuals with chronic health conditions.

Completer: *“I think it's very important. I think people should have access to food and different types of food that they normally would not be able to get at the store because of the prices of it, or just not knowing the different grains and different things out there. I think it's very important just in general to have services like that.” Female, age 45, employed part-time*

Non-completer: *“I think it's very important for people that, in my case, have trouble getting around, elderly people that they shouldn't be messing around the stove and everything, people [who are] disabled. It's very important.” Male, age 60, not employed*

Non-completer: *“Going through dialysis, if you come home from that, you're not going to want to cook. Any type of medical issues, someone that's disabled, or like I said, someone that's not as fortunate to be able to just work one job and then come home and then cook for their children… those are my top reasons right there, where it's a good service to have.” Male, age 59, unable to work*

Non-completers described the program overall as “good” and “useful,” but just not for them. Consistent with the positive attitudes they expressed, one of the main reasons for stopping the program was that they felt better and were able to cook for themselves again. Several participants wanted to make sure others who needed the program could take their place and benefit from it.

Non-completer: *“I'm now to a point where I can cook again. I can go to the store and buy my own groceries again. I can afford it again.” Female, age 53, employed full-time*

Non-completer: *“I just didn't want to take away from someone else that could be using it because I was back to my old self where I could cook on my own to keep myself busy, too.” Male, age 59, unable to work*

Similarly, for several other non-completers, their circumstances changed such that they felt they no longer needed it. These circumstances included getting settled after having moved and having greater means to buy food; relatives stepping in to help with food shopping; and undergoing changes in appetite and foods they could eat because of medical and surgical treatments for obesity.

Non-completer: *“Well, because I was living in [another state] and when I moved here, it was tough in the beginning and even my financial situation was-- and my wife wasn't working yet and I needed some system…I got it for a couple months and now I’m kind of situated and that's why I stopped doing those deliveries.” Male, age 44, not employed*

Non-completer: *“Yes, it was a lot of food. I had to stop it because it was piling up in my [freezer]. I was having another problem with my digestive system. I had started that Ozempic, so it slows down your digestive system. I couldn't eat what I wanted to eat. I could only eat a certain amount.” Female, age 65, retired*

#### 3.3.2 Thoughts about the meals

Both completers and non-completers had some issues with the meals, and “bland” was often used to describe them. However, many completers adapted to the meals, often growing to like them:

Completer: *“…It was bland. It would just get overwhelming sometimes. I would always have to put seasoning on everything.” Male, age 63, not employed*

Completer: *“That was fine for me. I'm used to eating bland, but even though it was bland, it was still good.” Female, age 35, employed part-time*

Completer: *“I just didn't think I was going to like it…Yes, at first, I was originally scared, but as time went on and I got to eat more and more, I was like, ‘Wow.’” Male, age 52, unable to work*

Non-completers, on the other hand, were less able to adapt to the taste, which was another reason for stopping the program. They did not get used to the food, and they did not want it to go to waste.

Non-completer: *“The taste and the flavor could have been a little bit more desirable. Even though it was supposed to be non-salted or sodium, it still could have been-- I just didn't like the taste of the food ... Maybe somebody else would like it, but I didn't like it, and I didn't want to waste it. I didn't want to keep having to not eat it or discard it. I don't want to do that. That's why I stopped the program.” Male, age 60, not employed*

Non-completer: *“It was nice the way they sent it. It was easier, but it was just the taste. That's all. I couldn't deal with the taste. It had no flavor...It was just plain. It doesn't matter what you try to add to it, it was just bland.” Female, age 54, not employed*

Non-completer: *“Then I just noticed as the weeks go by, the food started not to taste that great to me. I just didn't want to have to keep throwing away the plates, the trays of food… I don't know, maybe it was because as the week's gone by and I didn't like the food, I ate less and less of it maybe, I don't know.” Female, age 25, student*

They sometimes blamed themselves for being picky eaters or said that their sense of taste was off because of medical treatment, such as chemotherapy.

Non-completer: *“I guess I thought I had a more willing palate, but apparently not as much as I thought.” Male, age 32, employed full-time*

Non-completer: *“It could have been my taste buds were off a little bit from the chemo too, obviously, because I've actually just started coming back over like a month ago, because that stuff does a number on you.” Male, age 59, unable to work*

Like the taste of the meals, portion size was an initial concern for both groups. Completers described recognizing that portion control was part of improving their health, and they adapted. They described paying more attention to satiety signals due to their experience with the meals.

Completer: *“The portion sizes are good because remember that I said at the beginning they teach you, have been teaching people to especially that need to eat less, different kinds of stuff like the vegetables, the meat, the rice, you eat the soup. It was great that they did it like that so we can learn that we don't need to eat so much, you have to eat a little portion of everything.” Female, age not disclosed, employed full-time*

Completer: *“It looks small, but if you actually eat everything there, it tends to fill you up. It just takes a little bit. There were certain ones that it was almost like half a meal [laughs], but they did [take] a little getting used to as far as size-wise.” Male, age 51, employed part-time*

Completer: *“I think they could have been a little bit bigger, but I think that it's okay because, like I said, it helps me keep my blood sugars more in control because of the portions. I like to eat a lot, unfortunately, [chuckles] so I really just-- The portions are good. I think they're good enough. A good way to eat, the sizes that they give you, I think it's healthier that way.” Female, age 44, unable to work*

While many non-completers also recognized the importance of portion sizes in improving health, several thought the meals were too small. Two non-completers described various “workarounds” so that they could eat more, such as eating two of the meals provided (lunch and dinner) as a single meal or supplementing the meals with other foods.

Non-completer: *“I don't know what to expect from anything like this, but it turned out to be one meal a day. You could either use it for lunch or dinner and your choice...I'm just a good old-fashioned country boy from up north, so I eat a lot of food. Even before the program or after the program, it's still, to me, the same amount of food, so the program was just something that added to your meals. I guess I should say the meals weren't big enough to survive on one meal for me.” Male, age 51, not employed*

Non-completer: *“[I told myself] you can have a glass of milk after that, or maybe later I would, like I said, cut the bread in slices, put a little bit of butter, have that, find other means to keep myself full.” Female, age 54, employed part-time*

#### 3.3.3 Sustaining change after the program

Both completers and non-completers said the program had motivated them to maintain the healthier eating patterns demonstrated during the program after the meals ended, feeling it had served as a catalyst for longer-term, beneficial changes in dietary intake.

Completer: *“I'm really careful of what I eat today…It didn't matter what I ate before I got involved with Community Servings. [Now] I would buy food that didn't have that much salt in it because of my heart condition, or what type of food was good for my bones to stay strong.” Male, age 63, not employed*

Completer: *“I think I've been doing great. For me, I learned to eat better, less, and add more vegetables in every food that we eat in the house. I continue trying to do the same thing like in my head, ‘I don't need this, I don't need that. I don't need to do this,’ so I continue controlling my weight the way that I’m supposed to be.” Female, age not disclosed, employed full-time*

Non-completer: *“I'm still taking that to heart even after I stopped is trying to think of healthier options and think ahead of time so that when it does come time to eat at the end of the day, I'm not just burnt out from work and just say, ‘Oh, I'll just have whatever.’ It helped in that aspect of making me plan out a little bit more in advance.” Male, age 32, employed full-time*

Non-completer: *“Since becoming a part of the program, I have not completely reverted back to my old, horrible eating ways. Like, ‘Oh, let's have a pint of ice cream, a pizza, and French fries for dinner before bed…’ Honestly, I don't think I've had any ice cream since becoming a part of the program.” Female, age 53, employed full-time*

Patients in both groups described how experiencing the meals provided knowledge that helped facilitate sustained dietary changes.

Completer: *“It changed my vision of seeing food. Before I see food, I want to eat everything. I didn’t eat one piece of meat, I want to eat two, three. This changed my vision, I only need one piece, one palm of rice, not the two or three spoons of rice. It showed me the vision to eat less would be better and healthier.” Female, age not disclosed, employed full-time*

Non-completer: *I think it's pretty much like you get educated. The food comes in, and you start seeing those things. I was like, ‘Oh, okay, I can eat this. That's healthy.’” Male, age 44, not employed*

While this theme arose in both groups, completers typically cited more specific terms and strategies to help sustain dietary change. For example, one completer described saving the program food containers and using them to help maintain appropriate portion sizes, and another took pictures of meals she thought she could readily replicate.

Completer: *“I look at the ingredients and I take pictures of some of them and I said, ‘This is something I can do. That tastes good. Oh, wow, this is actually good. It tastes healthy. It's healthy, it's good.’ It gives me ideas for the future when I'm cooking different types of meals. It's not always boring or the same thing, but I can cook different things that actually can taste good that is healthy.” Female, age 42, employed full-time*

Completer: *“The portion control. I saved some of the containers so I can measure out what I'm eating, and it works for me. It's like I'm getting the meals again, but I'm doing it myself. I'm using the same dish they gave me, if that makes any sense, so I can learn how to eat better for myself.” Female, age 55, unable to work*

A final common theme among completers was that the cost of healthier foods remained a barrier.

Completer: *“You can't afford to eat the same thing as when the program was running. Now, it's like one day yes, one day no. It's not the same thing as like-- It's not like the product I had in the program.” Male, age 63, employed part-time*

## 4 Discussion

This study provides a comprehensive evaluation of patients’ motivations to enroll in, complete, or withdraw from an MTM program. Several findings were notable. Both completers and non-completers were interested in participating to alleviate symptoms and regain physical function. Both expressed very positive attitudes toward the program in terms of their personal experience, logistics of enrollment and meal delivery, and the important role of the program in healthcare delivery and society. Even though the non-completers left the program earlier, both groups felt the program motivated them to sustain improvements to their diets after the program ended, and that experiencing the meals helped them do that.

Non-completers were generally more enthusiastic about the program prior to enrolling and more likely to have been motivated based on goals to lose weight and to improve their nutrition, while completers were more interested in alleviating financial strain, time, and physical challenges associated with meal preparation. While some of the medical conditions addressed by the MTMs are comorbid with overweight and obesity, the meals are not generally designed for weight loss, but for nutritional support for chronic conditions. This suggests a potential discordance between patient expectations versus clinical goals of the program—a discordance that could be addressed in advance through clearer expectations regarding weight loss. These results also suggest that emphasizing the benefits of alleviating strains related to finances, time, and the physical challenges of meal preparation could help with long-term adherence and completion.

Both completers and non-completers expressed a desire for more flavorful foods, a finding similar to those in another qualitative study of lung cancer patients’ experiences with an MTM (20). Despite more initial hesitation about participation than non-completers, completers were often better able to adapt to the taste and portion size of the meals. These results suggest certain program adjustments might enhance completion. For example, while Community Servings currently provides information on how to season meals (without salt), it may be helpful for programs to offer supplemental spices, sauces, and seasonings. It could also help to inform participants at the outset that they may find the meals bland or smaller compared to their habitual diets, but that many people come to like the taste and portion size over time. Patients could also receive training in satiety cues, which would also develop a skill that could sustain changes once the program ends. While all Community Servings participants are offered three sessions with an RDN, in our study, very few reported using the nutrition education component. Although the RDNs attempt to reach out to participants, the non-response rate is high; in recent data from Community Servings regarding participants overall, only 5% attended all three nutrition counseling sessions, while 62% did not attend any sessions. It may be important to find effective ways to promote these sessions, since they could help address many of the issues that participants described. These findings also suggest further integration of the program with the healthcare system. For example, in routine clinical encounters, healthcare providers could assess patient satisfaction with the program, evaluate ongoing needs, and provide information and support related to the program. This would require additional training on the program for healthcare providers.

Many non-completers withdrew due to what they felt were positive reasons: they felt better and were able to cook for themselves. In this case, when physical functioning and health have improved sufficiently, an early exit may be appropriate. This underscores the need for further research to determine the optimal duration of MTM programs and how to individualize this through standardized clinical assessments. It also suggests that alternative “Food is Medicine” models could be considered; for example, programs could be designed to transition patients to medically tailored groceries or produce prescription programs to further support their restoration to health and well-being once they can cook and shop for themselves.

Our study has several strengths. It elevates the voices of patients who directly experience MTM programs. It included a direct comparison of themes between completers and non-completers. Potential limitations should be considered. Qualitative research such as this cannot assure random sampling, and thus findings may vary in certain subsets of patients. In this study, we were unable to contact 37 of 107 (34.6%) eligible patients in the recruitment process, suggesting that our sample may underrepresent certain patients, such as those with unstable telephone service or who do not feel up to answering the phone. Furthermore, more non-completers than completers were unreachable (25 versus 12 of the 37) or declined to participate (21 versus 9 of 30 who declined), suggesting that the findings from our sample of non-completers may be less representative of the perspectives of other non-completers. Although the MTM program is similar to others provided across the country by established non-profits, consistent with the common accreditation standards of the national Food is Medicine Coalition (3), our findings may be less generalizable to large for-profit MTM vendors.

In conclusion, these new findings identify patient reasons for enrolling in, completing, and withdrawing from MTM programs, informing strategies to improve program retention. Such strategies may include emphasizing the benefits related to alleviating financial and time stress, addressing expectations about weight loss, taste, and portion size, and transitioning patients to different types of Food is Medicine programs to further support their restoration to health as they feel better. Our results also highlight the utility of qualitative studies of patient perception for improving the efficiency and effectiveness of MTM programs.

## Data Availability

The full interview guide and the codebook used for analysis are available upon request. Transcripts are not shared outside of the research team because of the specificity of the sample in terms of roles and geographic region and the potential for identification of study participants, even with names and other identifiers removed.

## References

[1] Downer S Berkowitz SA Harlan TS Olstad DL Mozaffarian D. Food is medicine: actions to integrate food and nutrition into healthcare. BMJ. (2020) 369:m2482. doi: 10.1136/bmj.m2482 32601089 PMC7322667

[2] Mozaffarian D Aspry E Garfield K Kris-Etherton P Seligman H Velardeet GP . Food is medicine strategies for nutrition security and cardiometabolic health equity: JACC state-of-the-art review. J Am Coll Cardiol. (2024) 83:843–64. doi: 10.1016/j.jacc.2023.12.023 38383100

[3] Food is Medicine Coalition. Food is medicine coalition accreditation program. (2024). Available online at: https://fimcoalition.org/wp-content/uploads/2024/03/FIMC_Accreditation-One-Pager.pdf (Accessed 31 October 2025)

[4] Berkowitz SA Terranova J Randall L Cranston KDB Waters DB Hsu J. Association between receipt of a medically tailored meal program and health care use. JAMA Intern Med. (2019) 179:786–93. doi: 10.1001/jamainternmed.2019.0198, 31009050 PMC6547148

[5] Palar K Napoles T Hufstedler LL Seligman H Hecht FM Madsen K . Comprehensive and medically appropriate food support is associated with improved HIV and diabetes health. J Urban Health. (2017) 94:87–99. doi: 10.1007/s11524-016-0129-7, 28097614 PMC5359179

[6] Belak L Owens C Smith M Calloway E Samnadda L Egwuogu H . The impact of medically tailored meals and nutrition therapy on biometric and dietary outcomes among food-insecure patients with congestive heart failure: a matched cohort study. BMC Nutr. (2022) 8:108. doi: 10.1186/s40795-022-00602-y, 36192812 PMC9528877

[7] McIntosh N Billingsley H Hummel SL Mills WL. Medically tailored meals in heart failure: a systematic review of the literature, 2013-2023. J Card Fail. (2024). 31:939–950. doi: 10.1016/j.cardfail.2024.10.446 39674490

[8] Berkowitz SA Terranova J Hill C Ajayi T Linsky T Tishler LW . Meal delivery programs reduce the use of costly health care in dually eligible Medicare and Medicaid beneficiaries. Health Aff (Millwood). (2018) 37:535–42. doi: 10.1377/hlthaff.2017.0999, 29608345 PMC6324546

[9] Gurvey J Rand K Daugherty S Dinger C Schmeling J Laverty N. Examining health care costs among MANNA clients and a comparison group. J Prim Care Community Health. (2013) 4:311–7. doi: 10.1177/2150131913490737, 23799677

[10] Palar K Sheira LA Frongillo EA O'Donnell AA Napoles TM Ryle M . Food is medicine for human immunodeficiency virus: improved health and hospitalizations in the changing health through food support (CHEFS-HIV) pragmatic randomized trial. J Infect Dis. (2025) 231:573–82. doi: 10.1093/infdis/jiae195, 38696724 PMC11911788

[11] Berkowitz SA Delahanty LM Terranova K Steiner B Ruazol MP Singh R . Medically tailored meal delivery for diabetes patients with food insecurity: a randomized cross-over trial. J Gen Intern Med. (2019) 34:396–404. doi: 10.1007/s11606-018-4716-z 30421335 PMC6420590

[12] Tapper EB Baki J Nikirk S Hummel S Asrani SK Lok AS. Medically tailored meals for the management of symptomatic ascites: the SALTYFOOD pilot randomized clinical trial. Gastroenterol Rep (Oxf). (2020) 8:453–6. doi: 10.1093/gastro/goaa059, 33442478 PMC7793123

[13] Tapper EB Saleh ZM Nikirk S Bajaj J Chen X Lok AS. Medically tailored meals for patients with cirrhosis and hepatic encephalopathy: the BRAINFOOD proof-of-concept trial. J Clin Exp Hepatol. (2024) 14:101439. doi: 10.1016/j.jceh.2024.101439, 38882178 PMC11176801

[14] Go AS Tan TC Horiuchi KM Laws D Ambrosy AP Leeet KK . Effect of medically tailored meals on clinical outcomes in recently hospitalized high-risk adults. Med Care. (2022) 60:750–8. doi: 10.1097/MLR.0000000000001759 35972131 PMC9451942

[15] Compher C Henstenburg A Aloupis M Sun A Quinn R Emery E . The nutritional impact of 7 versus 21 home-delivered medically tailored meals in patients with heart failure and malnutrition risk: a random order crossover feeding trial (MEDIMEALS). BMC Nutr. (2025) 11:56. doi: 10.1186/s40795-025-01036-y, 40102963 PMC11916996

[16] Woodward EN Matthieu MM Uchendu US Rogal S Kirchner JE. The health equity implementation framework: proposal and preliminary study of hepatitis C virus treatment. Implement Sci. (2019) 14:26. doi: 10.1186/s13012-019-0861-y, 30866982 PMC6417278

[17] Woodward EN Singh RS Ndebele-Ngwenya P Melgar Castillo A Dickson KS Kirchner JE. A more practical guide to incorporating health equity domains in implementation determinant frameworks. Implement Sci Commun. (2021) 2:61. doi: 10.1186/s43058-021-00146-5, 34090524 PMC8178842

[18] Hsieh HF Shannon SE. Three approaches to qualitative content analysis. Qual Health Res. (2005) 15:1277–88. doi: 10.1177/1049732305276687, 16204405

[19] Landis JR Koch GG. The measurement of observer agreement for categorical data. Biometrics. (1977) 33:159–74. doi: 10.2307/2529310 843571

[20] Owens CE Keaver L Chandiramani D McCann K Cohen MK Chandra J . Medically tailored meals in lung cancer care: patient experiences from the nutricare clinical trial. J Cancer Surviv. (2025). doi: 10.1007/s11764-025-01857-7 40643889

